# Tumor Cell Phenotype Is Sustained by Selective MAPK Oxidation in Mitochondria

**DOI:** 10.1371/journal.pone.0002379

**Published:** 2008-06-11

**Authors:** Soledad Galli, Valeria Gabriela Antico Arciuch, Cecilia Poderoso, Daniela Paola Converso, Qiongqiong Zhou, Elisa Bal de Kier Joffé, Enrique Cadenas, Jorge Boczkowski, María Cecilia Carreras, Juan José Poderoso

**Affiliations:** 1 Laboratory of Oxygen Metabolism, University Hospital, University of Buenos Aires, Buenos Aires, Argentina; 2 Department of Human Biochemistry, Faculty of Medicine, University of Buenos Aires, Buenos Aires, Argentina; 3 Department of Pharmacology and Pharmaceutical Sciences, School of Pharmacy, University of Southern California, Los Angeles, California, United States of America; 4 Institute of Oncology Ángel H. Roffo, University of Buenos Aires, Buenos Aires, Argentina; 5 Unité INSERM 700, Hôpital Bichat, Paris, France; 6 Department of Clinical Biochemistry, School of Pharmacy and Biochemistry, University of Buenos Aires, Buenos Aires, Argentina; 7 Department of Medicine, University Hospital, University of Buenos Aires, Buenos Aires, Argentina; University of Oldenburg, Germany

## Abstract

Mitochondria are major cellular sources of hydrogen peroxide (H_2_O_2_), the production of which is modulated by oxygen availability and the mitochondrial energy state. An increase of steady-state cell H_2_O_2_ concentration is able to control the transition from proliferating to quiescent phenotypes and to signal the end of proliferation; in tumor cells thereby, low H_2_O_2_ due to defective mitochondrial metabolism can contribute to sustain proliferation. Mitogen-activated protein kinases (MAPKs) orchestrate signal transduction and recent data indicate that are present in mitochondria and regulated by the redox state. On these bases, we investigated the mechanistic connection of tumor mitochondrial dysfunction, H_2_O_2_ yield, and activation of MAPKs in LP07 murine tumor cells with confocal microscopy, *in vivo* imaging and directed mutagenesis. Two redox conditions were examined: low 1 µM H_2_O_2_ increased cell proliferation in ERK1/2-dependent manner whereas high 50 µM H_2_O_2_ arrested cell cycle by p38 and JNK1/2 activation. Regarding the experimental conditions as a three-compartment model (mitochondria, cytosol, and nuclei), the different responses depended on MAPKs preferential traffic to mitochondria, where a selective activation of either ERK1/2 or p38-JNK1/2 by co-localized upstream kinases (MAPKKs) facilitated their further passage to nuclei. As assessed by mass spectra, MAPKs activation and efficient binding to cognate MAPKKs resulted from oxidation of conserved ERK1/2 or p38-JNK1/2 cysteine domains to sulfinic and sulfonic acids at a definite H_2_O_2_ level. Like this, high H_2_O_2_ or directed mutation of redox-sensitive ERK2 Cys^214^ impeded binding to MEK1/2, caused ERK2 retention in mitochondria and restricted shuttle to nuclei. It is surmised that selective cysteine oxidations adjust the electrostatic forces that participate in a particular MAPK-MAPKK interaction. Considering that tumor mitochondria are dysfunctional, their inability to increase H_2_O_2_ yield should disrupt synchronized MAPK oxidations and the regulation of cell cycle leading cells to remain in a proliferating phenotype.

## Introduction

The cell's redox status controls the progression of the cell cycle, including misregulation in cancer [1], [2]. Oxidants, such as H_2_O_2_, play an important role in the activation of signaling molecules, which control the complex machinery involved in cell proliferation, differentiation, apoptosis, and senescence. An attractive notion is that the continuous increase in oxidant concentration may trigger disparate cell responses: slight variations in H_2_O_2_ concentration (0.7–20 µM H_2_O_2_) help determine normal cell fate, *i.e.,* proliferation [3], [4], arrest, senescence or apoptosis [5]. Moreover, an increase in H_2_O_2_ steady-state concentration ([H_2_O_2_]_ss_) has been observed *in vivo* in the transition from proliferative hepatoblasts to quiescent and differentiated hepatocytes [6].

Mitochondria are major cellular sources of H_2_O_2_, the production of which is modulated by the mitochondrial energy state and generation of nitric oxide [7]. High mitochondrial H_2_O_2_ yield is associated with late rat brain and liver development and signals the end of proliferation [6], [8]. From this perspective, development can be understood as a transition from anaerobic metabolism to a five-fold increase in metabolism in mature cells; arrest and differentiation are associated to high mitochondrial activity and membrane potential [9]. Mitochondria are dysfunctional in cancer: the activity of mitochondrial complexes is decreased, the mitochondrial generation of H_2_O_2_ is substantially decreased [10], the mitochondrial-K^+^ channel axis is suppressed [11], the oxidant-dependent inhibition p38 MAPK is impaired, and p53 suppresses mitochondrion-driven apoptosis [12]. Hence, it may be surmised that tumor cells –alike embryonic tissues– live at a very low [H_2_O_2_]_ss_ [6], [10], [13].

Signal transduction is often orchestrated by mitogen-activated protein kinases (MAPKs) [14]. MAPKs are proline-directed serine/threonine kinases [15] that have been classified into at least six subfamilies; from these, ERK1/2, JNK1/2, and p38 are the most extensively studied. ERK1/2 is normally activated by growth signals [16], [17]; JNK1/2 and p38 respond to oxidative stress, heat shock, ionizing radiation, and UV light [18], [19], and are mainly associated with cell cycle arrest and apoptosis. Of note, oxidative stress may be viewed as a potential carcinogen due to the activation of NFκB or Akt pathways or by causing DNA mutations [20], [21]. MAPKs are specifically regulated by a MAPK kinase (MAPKK) [22], *i.e.,* ERK1/2 is activated by MEK1/2, p38 by MKK3, and JNK1/2 by MKK4, among others. MAPKs are sensitive to redox changes: ERK1/2, p38, and JNK1/2 are activated in a variety of cellular systems at different H_2_O_2_ concentrations [23], [24]. We previously reported that high phosphorylated ERK1/2 content is associated with proliferation and low [H_2_O_2_]_ss_ in proliferating embryonic and tumoral tissues, while tumor arrest requires high [H_2_O_2_]_ss_ with predominant p38 and JNK1/2 activation [6], [10].

To understand the mechanisms of redox modulation by MAPKs, studies have been focused on the oxidative inhibition of phosphatases (MKP) [25] and on the interaction of MAPKKs with antioxidant proteins, such as thioredoxin [26]. However, the direct effects of oxidants on MAPKs and the mechanisms of the transition from proliferation to arrest remain obscure.

Recent data indicate that MAPKs are present in mitochondria, as well as other kinases like PKC and Akt [27], [28]. However, the connection among tumor mitochondrial dysfunction, H_2_O_2_ yield, and activation of MAPKs still awaits elucidation. In the present work, we provide evidence of MAPKs subcellular redistribution upon activation, including their transit through mitochondria. We demonstrate that the redox state modulates the mitochondrial interaction of MAPKs to MAPKKs by oxidation of conserved cysteine domains of MAPKs to sulfinic and sulfonic acid; this biochemical mechanism determines MAPKs differential activation and traffic to nuclei and ultimately, sustains the phenotype of LP07 tumor cells.

## Results

### H_2_O_2_ drives opposite effects on tumor cell cycle progression and signaling

**Figure 1:**
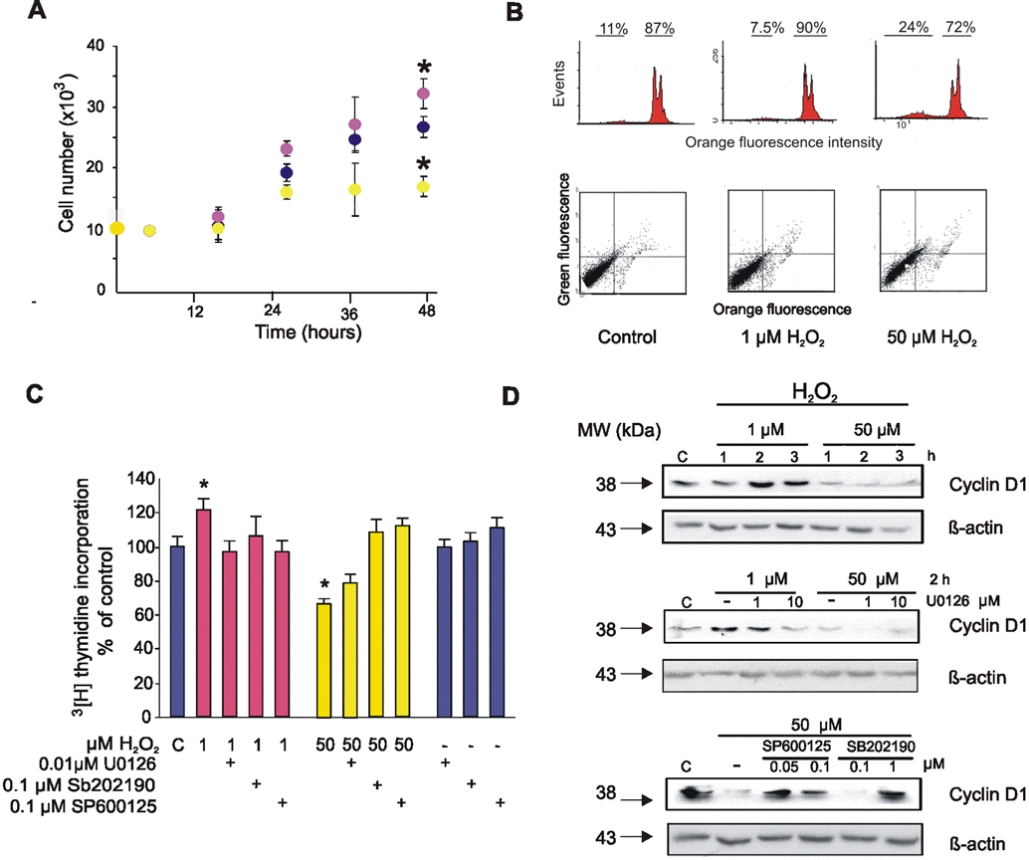
Redox dual modulation of cell fate depends on the activation of ERK1/2 or p38 and JNK1/2. (A) Cells were counted up to 48 h after stimulation with 1 µM (purple), 50 µM (yellow) or no H_2_O_2_ (blue) (mean±s.e.m; n = 3, experiment representative of 3, * *p*<0.05 respect to control values by ANOVA and Scheffe comparison test). (B) Apoptosis was determined by propidium iodide staining (upper panel) and Annexin V (lower panel) by flow cytometry 48 h after H_2_O_2_ treatment. (C) [^3^H] thymidine incorporation was measured 48 h after supplementing LP07 cells with 1 µM (purple), 50 µM (yellow) or no H_2_O_2_ (blue) (C = control) (mean±s.e.m; n = 8, experiment representative of 5, *p<0.05 respect to control values by ANOVA and Scheffe comparison test). When appropriate, cells were preincubated 2 h prior to stimulation with ERK1/2 (U0126), p38 (SB202190) or JNK1/2 (SP600125) inhibitors. (D) Cyclin D1 expression was determined 1 to 3 h after H_2_O_2_ treatment (upper panels), and 2 h after stimulation in the presence of MAPK inhibitors as in (C) (medium and lower panels).

P07 tumor cells show very low [H_2_O_2_]_ss_ (10^−11^ M) like embryonic and proliferating tissues [6], [10]. In these cells, mitochondria have a low H_2_O_2_ production rate but they still respond to oxidative stress as the normal ones do [6], [10]. In the present study, we thus examined the redox transition as represented by low (1 µM) and high (50 µM) H_2_O_2_ concentrations. This transition offers the opportunity to test a) the circuit of redox signaling based upon mitochondria and, b) a mechanistic of low H_2_O_2_ yield for persistent cell proliferation. We show in Figure 1A that 1 µM H_2_O_2_ increased LP07 cell proliferation by about 20% (*p<.05)* while 50 µM H_2_O_2_ oppositely resulted in 40% decrease of cell proliferation (*p<.05*). To evaluate if 50 µM H_2_O_2_ caused cell cycle arrest or apoptosis, we performed Annexin V staining by flow cytometry. Annexin V-propidium iodide double-negative cells indicated that high H_2_O_2_ concentration triggered a transition to a low proliferative state but not to apoptosis, as plotted in Fig. 1B.

The cell cycle modulation by H_2_O_2_ described herein was orchestrated by MAPKs. At low H_2_O_2_, redox-induced cell proliferation was almost abolished by ERK1/2 inhibitor U0126. Instead, cell cycle arrest observed with 50 µM H_2_O_2_ was specifically mediated by activation of p38 and JNK1/2 as shown in Fig. 1C by utilizing SB202129 (p38 inhibitor) or SP600125 (JNK1/2 inhibitor). As inferred by the effect of U0126, the temporal increase of cyclin D1 observed with 1 µM H_2_O_2_ depended as well on the activation of ERK1/2, while downregulation of cyclin D1 at 50 µM of H_2_O_2_ was reverted by the p38 and JNK1/2 inhibitors (Fig. 1C and D). It is concluded that cell cycle variations are related to the differential redox activation of extracellular or stress activated MAPK.

### Mitochondria are a meeting point of MAPKs and their upstream activators

**Figure 2:**
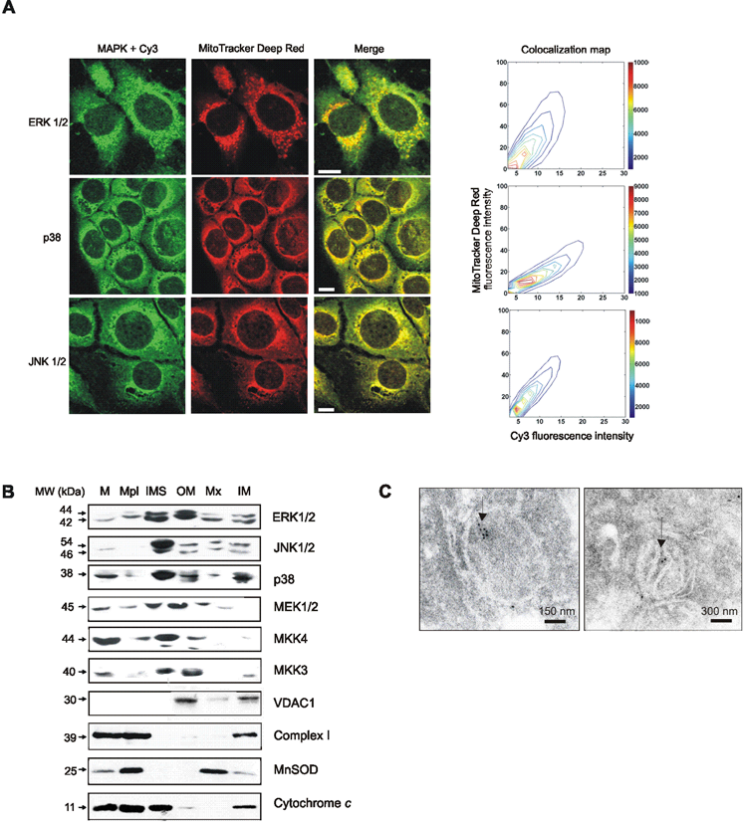
MAPKs and MAPKKs localize in tumor mitochondria. (A) LP07 cells were stained with MitoTracker Deep Red, fixed and immune stained with anti ERK1/2, JNK1/2 and p38 primary antibodies and secondary antibodies conjugated with Cy3, and analyzed in an Olympus FV1000 confocal microscope. Images directly exported from Olympus Fluoview acquisition program were processed with DIP image software for MATLAB, and a 2D fluorescence intensity histogram was performed. Pixel frequency map displayed on the right. Bar = 10 µm. (B) Submitochondrial localization of MAPKs and MAPKKs was assessed by western blot. (M: mitochondria; Mpl: mitoplast; OMM: outer mitochondrial membrane; IMS: intermembrane space; IMM: inner mitochondrial membrane; Mx: mitochondrial matrix). Identity of mitochondrial fractions was corroborated with specific antibodies anti complex I 39 kDa subunit, voltage-dependent anion channel (VDAC1), superoxide dismutase II and cytochrome oxidase, subunit VI C. (C) ERK1/2 was detected in LP07 mitochondria by immune labelling and transmission electron microscopy.

Activation of MAPKs in response to a variety of stimuli, including oxidative stress [19], [29] has been defined in a two-compartment system: phosphorylation (activation) in cytosol followed by translocation to nuclei [16], [30]. Preliminary data on MAPK mitochondrial turnover and its modulation by redox status [24], [31] prompted us to examine a three-compartment model for MAPK redistribution upon activation in LP07 cells. To confirm the presence of MAPKs in LP07, cells were labeled with MitoTracker Deep Red and immune stained with anti ERK1/2, p38, and JNK1/2 primary antibodies and secondary antibodies conjugated with Cy3. Images were obtained by confocal microscopy and further analyzed by intensity correlation analysis (ICA) [32] (Fig. 2A); the presence of a diagonal in the 2D fluorescence intensity histogram demonstrated that MAPKs are constitutively expressed in mitochondria. Furthermore, mitochondrial subfractionation showed that MAPKs colocalized with their cognates MEK1/2, MKK3 and MKK4 in the mitochondrial outer membrane and in the intermembrane space (Fig. 2B), as corroborated by the different fraction markers. ERK1/2 molecules were also detected within the organelles by electron microscopy (Fig. 2C).

### Traffic of MAPKs and MAPKKs elicited by redox stimulation involves the mitochondrial compartment

**Figure 3:**
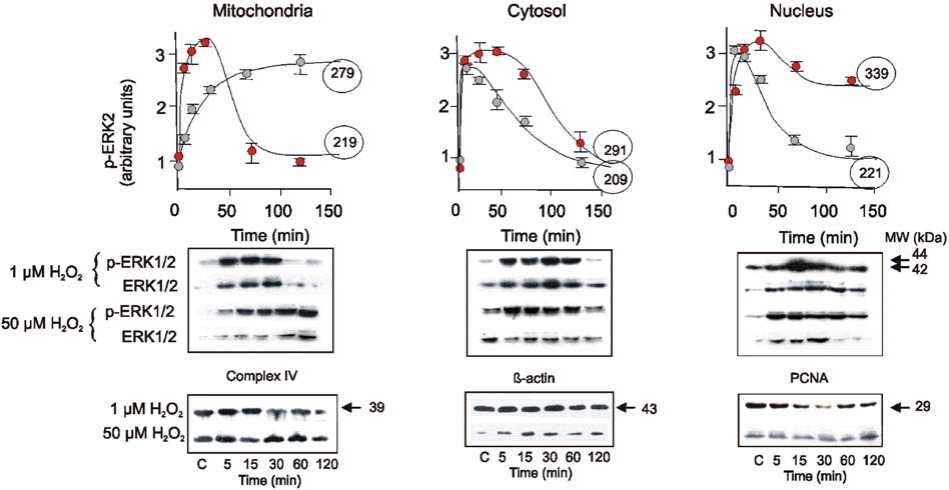
Kinetics of ERK1/2 activation and subcellular redistribution upon redox stimuli. Temporal activation and distribution of ERK1/2 and p-ERK1/2 in the subcellular fractions was followed by western blot. Red and grey circles correspond to 1 and 50 µM H_2_O_2_, respectively; each point integrates densitometries from three separate experiments. Circled numbers represent areas under the curve in arbitrary units per minute calculated with Graph Pad Prism 5 software. A western blot representative of 3 independent experiments is shown. Protein loading was determined with antibodies anti cytochrome oxidase subunit VI C for mitochondria, β-actin for cytosol, and nuclear antigen (PCNA) for nuclei.

Comparison of the activation kinetics and nuclear accumulation of ERK1/2, JNK1/2, and p38 upon oxidative perturbation yielded a remarkable difference in MAPK responses. After stimulation with 1 µM H_2_O_2_, p-ERK1/2 and total ERK1/2 increased in mitochondria, cytosol and nuclei (Fig. 3). An hour after stimulation, p-ERK1/2 returned to the basal level in mitochondria but remained elevated in nuclei. Conversely, 50 µM H_2_O_2_ entailed a considerable lower rate of ERK1/2 translocation and reduced its activation by ten-fold, and the kinase was retained in mitochondria in detriment of nuclear accumulation (Fig. 3). The sum of the kinetics integrals for each H_2_O_2_ treatment remained constant (Fig. 3, circled numbers) which emphasizes the notion of redox interdependence between the mitochondrial and nuclear compartments.

**Figure 4:**
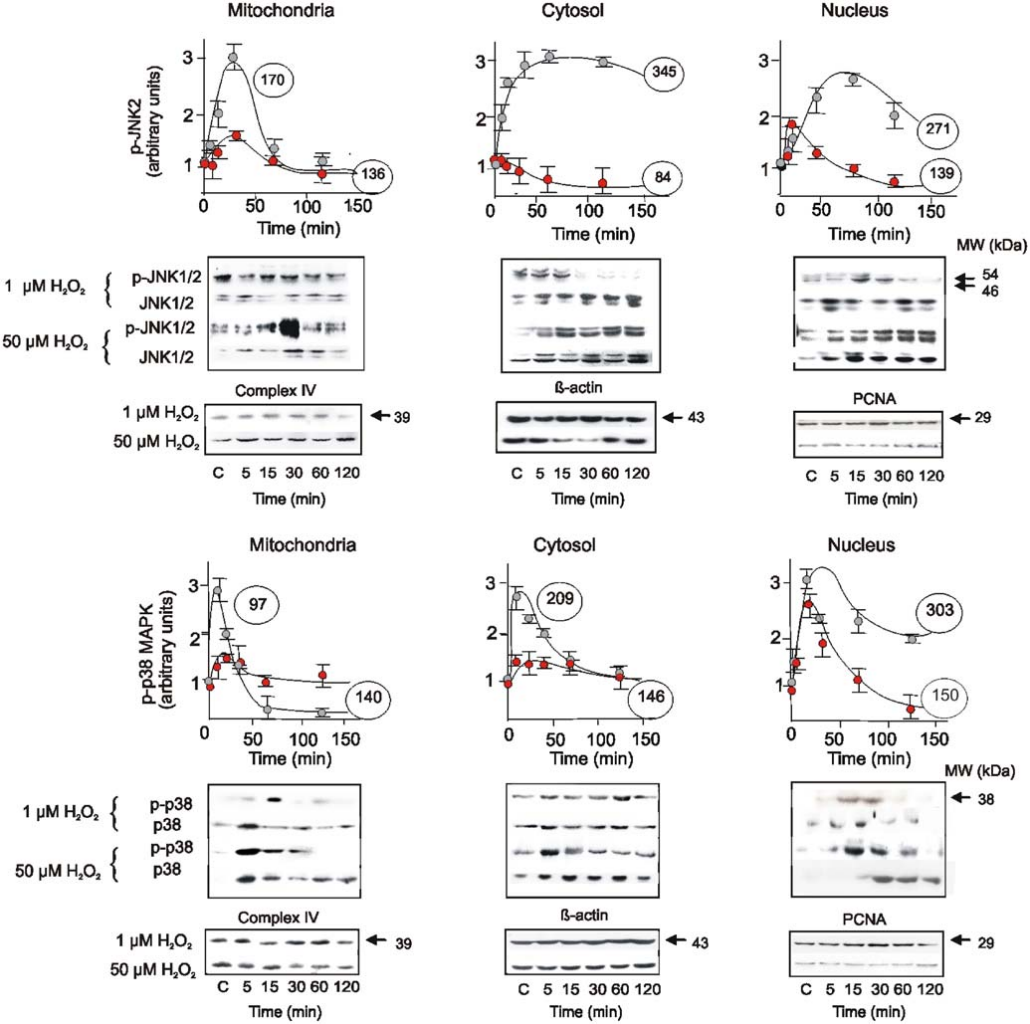
Kinetics of JNK1/2 and p38 activation and subcellular redistribution upon redox stimuli. Temporal activation and distribution of JNK1/2 and p38 was followed as in Fig. 3, in analogue experimental conditions.

A similar approach to assess the redox regulation of the kinetics JNK1/2 and p38 (Fig. 4) revealed that these were barely affected at low (1 µM) H_2_O_2_ levels. Instead, upon treatment with 50 µM H_2_O_2_, total and phosphorylated JNK1/2 and p38 increased in mitochondria and cytosol and then translocated and accumulated in the nuclei (Fig. 4).

**Figure 5:**
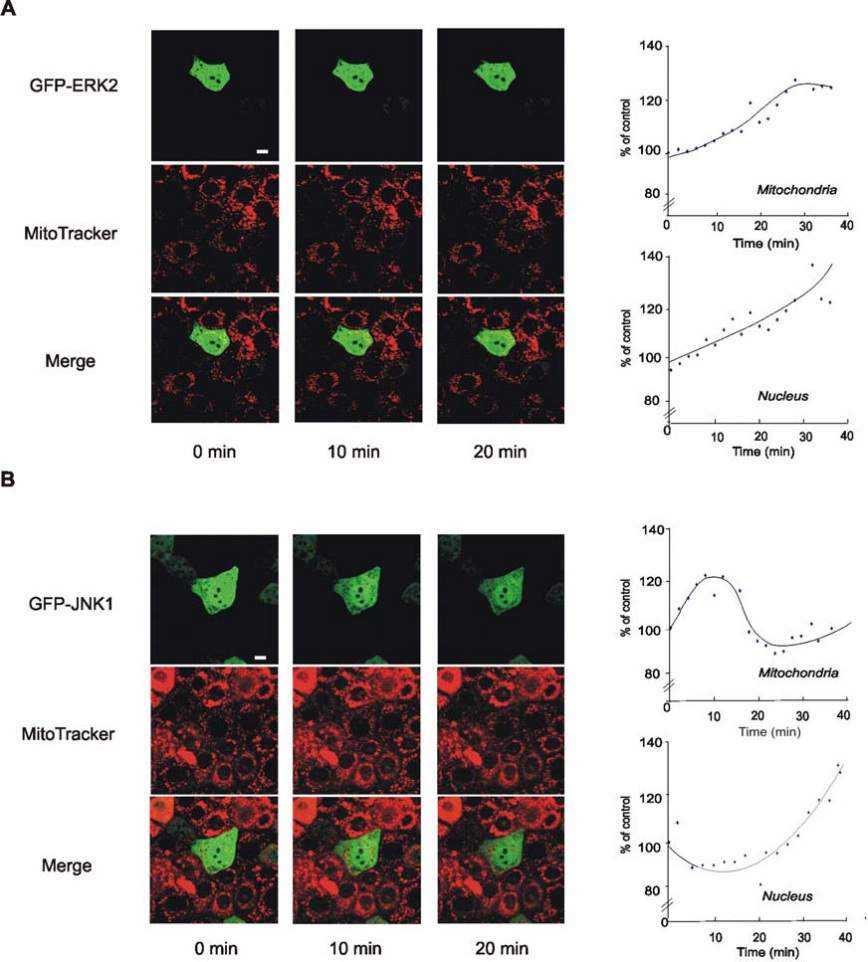
Imaging of MAPKs *in vivo*. (A) LP07 cells stained with MitoTracker Deep Red and transfected with GFP-hERK2 and stimulated with 1 µM H_2_O_2_, or (B) transfected with GFP-JNK1 and stimulated with 50 µM H_2_O_2_ were imaged *in vivo.* In (A) and (B) on the right, turnover of MAPKs was monitored with a mask done on the mitochondria with the MitoTracker, or by appearance of GFP green fluorescence in the nucleus. Changes of fluorescence were graphicated when MitoTracker intensity and the respective GFP fluorescence were over 20 in these areas. Images were taken every minute for 40 minutes. Bar = 10 µm.

In order to assess the *in vivo* translocation of MAPKs into mitochondria, LP07 cells were transfected with either GFP-hERK2 or GFP-hJNK1, stained them with MitoTracker Deep Red, and continuously followed for MAPK redistribution upon H_2_O_2_ stimulation by video confocal microscopy. Low H_2_O_2_ caused GFP-hERK2 entrance to mitochondria and subsequent translocation to nuclei (Fig. 5A). A similar behaviour but at high H_2_O_2_ stimulation was found for GFP-hJNK1 (Fig. 5B). In the right panel of Figure 5, fluorescence values were plotted every minute during 40 min. In both redox conditions, a previous passage to mitochondria anticipated further traffic to nucleus; however, in the experimental conditions, GFP-hJNK1 turnover resulted faster than that of GFP-hERK2. The patterns described by confocal microscopy were similar to those observed by western blot in Figs. 3 and 4.

The subcellular redistribution of MAPKKs and their redox regulation are shown in Fig. S1. 1 µM H_2_O_2_ stimulus elicited an initial MEK1/2 outward movement from mitochondria to cytosol (Fig. S1A). As ERK1/2, p-MEK1/2 was retained in mitochondria when stimulated with 50 µM H_2_O_2_. MEK1/2 translocation to nuclei could not be detected in agreement with previous reports [29], [33].

The pattern of redistribution and activation of MKK4 (Fig. S1B) and MKK3 (Fig. S1C) following stimulation with high H_2_O_2_ mimicked that of MEK1/2 at 1 µM H_2_O_2_, i.e., the MAPKKs moved out from the mitochondria to the cytosol. Fig. S1D is a scheme representative of putative cycle of upstream MAPKKs in the studied conditions to carry up MAPKs to nucleus. The degree of MAPK nuclear retention is related to cell cycle progression [16], [33]; ERK1/2 retention correlates to cell proliferation, while retention of JNK1/2 and p38 correlates with cell cycle arrest (see Figs. 1 and 2).

### Oxidation favors differential MAPKs phosphorylation in vitro, but does not change the intrinsic catalytic activity

**Figure 6:**
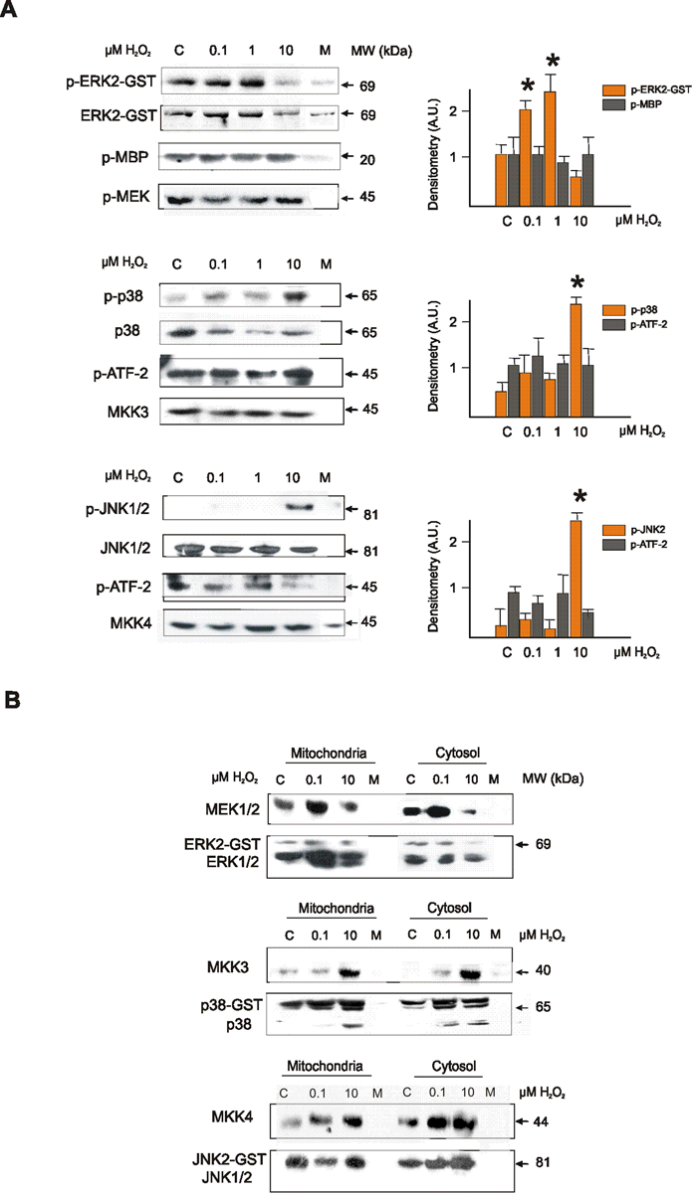
Redox state modulates MAPKs phosphorylation through differential binding to MAPKKs *in vitro*. (A) hERK2-GST, or immuneprecipitated JNK1/2 and p38 were oxidized for 15 min with H_2_O_2_ and then incubated 30 min with their upstream kinases (MEK1/2, MKK4 or MKK3, respectively), or their substrates, myelin basic protein (MBP) or ATF-2, respectively, in the presence of ^32^P-γATP. Total protein content for MAPKKs and MAPKs assessed by western blot. Mock experiment (M) was done as control in which the phosphorylating kinases (MAPKK or MAPK, respectively) were omitted. Asterisk denotes *p*<0.05 respect to control values by ANOVA and Scheffe comparison test. (B) Human recombinant ERK2-GST, JNK2-GST or p38-GST were immobilized on agarose, oxidized with H_2_O_2_ and incubated with cytosolic or mitochondrial fractions. Interaction with MAPKKs was detected by western blot.

Direct effects of H_2_O_2_ on MAPK catalytic activity were assessed with human recombinant ERK2-GST or immunoprecipitated JNK1/2 and p38 immobilized on agarose. Kinases exposed to H_2_O_2_ were subsequently incubated with substrates or upstream kinases and ^32^P-γATP. Phosphorylation efficiency of ERK2 by MEK1/2 was enhanced at low H_2_O_2_ concentrations, whereas decreased at high H_2_O_2_ concentration (Fig. 6A). Oppositely, phosphorylation of JNK1/2 and p38 by respective MKK4 and MKK3, was enhanced at high H_2_O_2_ level (Fig. 6A). However, H_2_O_2_ treatments did not affect the intrinsic catalytic activity of ERK2, JNK1/2, or p38 as proved by absence of effects on the phosphorylation of myelin basic protein (substrate for ERK1/2) or ATF-2 (substrate for p38 and JNK1/2) (Fig. 6A). We therefore surmise that exposure of ERK2, JNK1/2, and p38 to determined H_2_O_2_ concentrations increases phosphorylation efficiency by introducing a post-translational modification that would enhance the interaction with their respective MAPKKs.

To see whether phosphorylation efficiency varies by redox effects on binding, we treated recombinant MAPKs with 0.1–10 µM H_2_O_2_, and subsequently incubated them with cytosolic or mitochondrial fractions containing the respective MAPKKs. Similarly to phosphorylating activities, ERK2-GST binding to MEK1/2 resulted enhanced at low H_2_O_2_ level (0.1 µM H_2_O_2_) (Fig. 6B) whereas p38-GST and JNK2-GST binding to cognate MAPKKs was facilitated at 10 µM H_2_O_2_ (Fig. 6B). Interestingly, oxidation of ERK2-GST enhanced its dimerization and activation as shown by the interaction with endogenous ERK1/2 and the increase in phosphorylation (Fig. 6B). However, these interactions and ERK2 activation were enterely disrupted at H_2_O_2_ concentrations above 1 µM.

### H_2_O_2_ modulates ERK-MEK interaction and shuttle to nuclei

**Figure 7:**
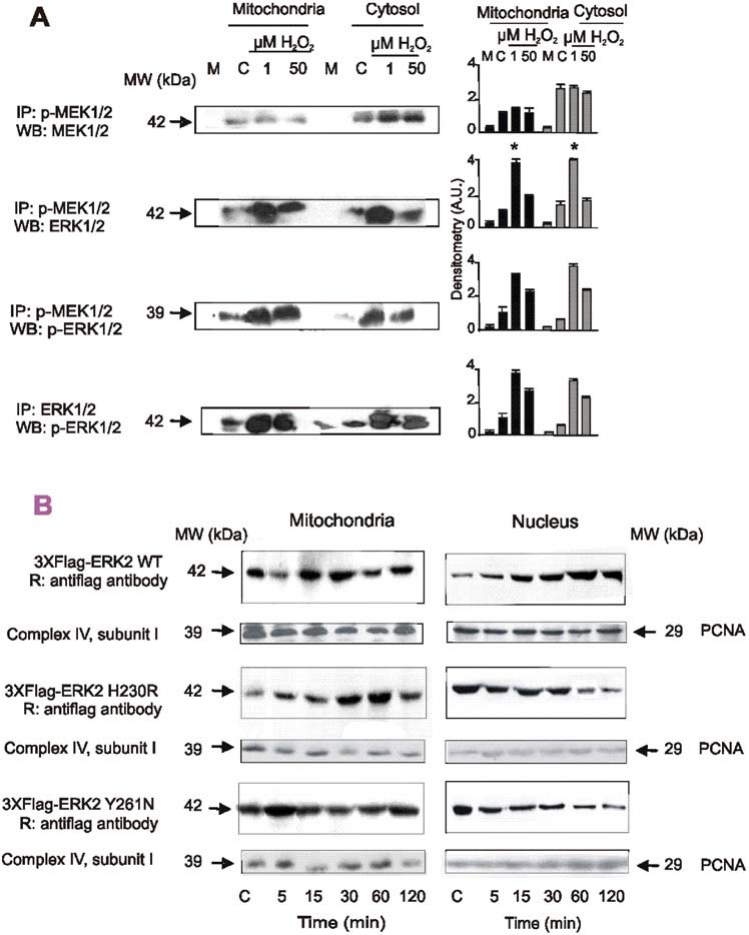
Redox state adjusts MEK-ERK interaction and regulates traffic from mitochondria to nuclei *in vivo*. (A) p-MEK1/2 and p-ERK1/2 were immunoprecipitated from mitochondrial or cytosolic extracts of H_2_O_2_ stimulated cells, run on SDS-PAGE and stained with anti MEK1/2, p-MEK1/2, ERK1/2 and p-ERK1/2 antibodies; bars are mean±s.e.m.; n = 3, western blot representative of 3 independent experiments displayed on the left, and densitometries on the right. Asterisk denotes *p*<0.05 respect to control values by ANOVA and Scheffe comparison test. (B) Cells were transfected with wild type (WT) ERK2, or ERK2 mutants H230R or Y261N, both with reduced binding capacity to MEK1/2 [33]. After incubation with H_2_O_2_, mitochondrial and nuclear fractions were isolated, and ERK2 distribution was detected by western blot using an anti Flag antibody. A western blot representative of 3 independent experiments is shown. Complex IV and PCNA were used as loading control.

To examine whether redox effects on binding *in vivo* resemble the ones observed *in vitro,* LP07 cells were stimulated with 1 and 50 µM H_2_O_2_, p-MEK1/2 and ERK1/2 were precipitated from mitochondria, and cytosol and complex formation was followed by western blot in a pull-down assay. As observed in the *in vitro* assay, *in vivo* p-MEK1/2-ERK1/2 interaction was substantially increased at low H_2_O_2_ and decreased at high H_2_O_2_ concentration (Fig. 7A).

To investigate whether modulation of MEK-ERK interaction in mitochondria affects shuttle to nuclei, cells were transfected with ERK2 and its mutants H230R or Y261N, both with restricted docking to MEK1/2 [33]. At low H_2_O_2,_ transfected ERK2 wild type followed a typical sequence of translocation to mitochondria and nucleus as shown in Figures 2 and 5A. Oppositely, ERK2 mutants with poor binding to MEK1/2 were retained in the organelles in detriment of their translocation to nuclei (Fig. 7B). These findings suggest that the traffic of MAPKs to the nuclei and thus, cell behaviour, depend on H_2_O_2_-induced changes in their loop of activation, as resulted from redox variations in the domains docking the upstream MAPKKs in mitochondria [34], [35].

### ERK2, p38, and JNK2 cysteine thiols are specifically oxidized by H_2_O_2_

**Figure 8:**
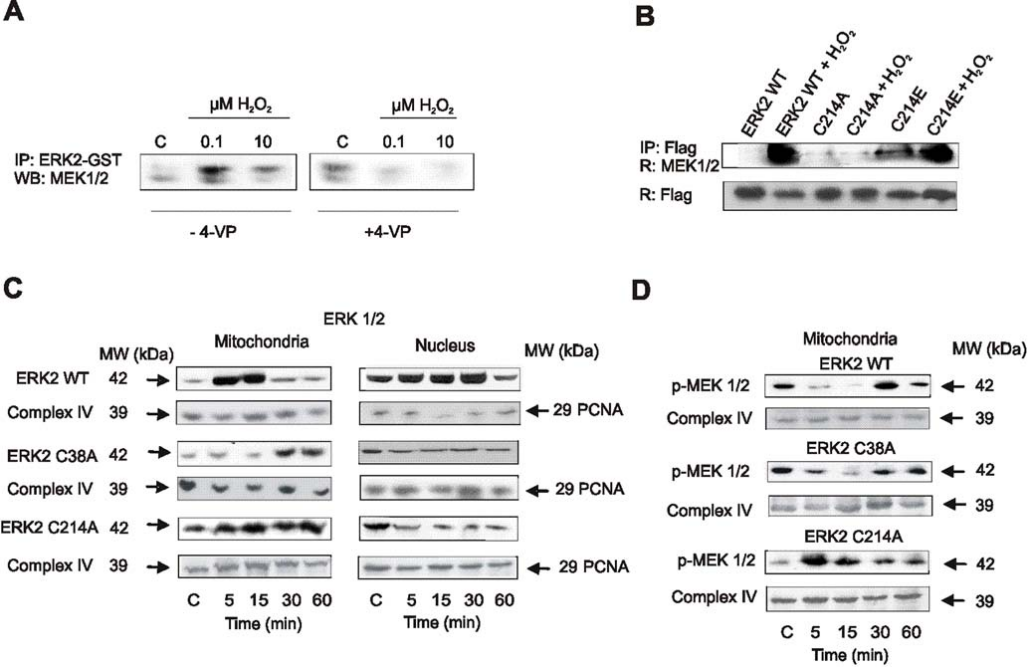
ERK interaction with MEK and efficient shuttle to nuclei critically depend on the oxidation of ERK Cys^38^ and Cys^214^. (A) hERK2-GST cysteines were blocked with 4-vinylpyridine (4-VP), and the protein oxidized and subsequently incubated with a mitochondrial fraction as in Fig. 8. (B) Cells were transfected with wild type (WT) ERK2 or ERK2 mutants C38A, C214A, and C214E and stimulated with 1 µM H_2_O_2_ for 15 min. ERK2 was immunoprecipitated with an anti Flag antibody from isolated mitochondria, and complexes were run on SDS-PAGE and stained with an anti MEK1/2 antibody. (C) Kinetics of differential mitochondrial and nuclear distribution of transfected wild type and ERK2 mutants were followed by western blot after 1 µM H_2_O_2_ treatment. (D) Kinetics of mitochondrial distribution of p-MEK1/2 when cells were transfected with ERK2 WT, C38A or C214A.

Considering the susceptibility to oxidation of cysteine thiol moieties in proteins, we explored the relevance of these amino acids in the regulation of MAPKs pathways. ERK2-GST immobilized on agarose was exposed to the thiol blocker 4-vynil-pyridine (4-VP) and then incubated with a mitochondrial fraction. 4-vinylpyridine treatment resulted in a markedly reduced ERK-MEK interaction (Fig. 8A).

Oxidized cysteines responsible for the differential binding of MAPKs were identified by LC/MS/MS. After treatment with low H_2_O_2_ concentrations (0.1 µM), the thiol groups of ERK2 Cys^38^ and Cys^214^ were oxidized to sulfinic (–SO_2_H) and sulfonic acid (–SO_3_H). No oxidation of ERK2 cysteines was detected following treatment with high H_2_O_2_ concentrations (10 µM) (Table 1).

**Table 1:** Mass spectrometry analysis of ERK2, p38 and JNK2 cysteine modification by H_2_O_2_

MAPK	H_2_O_2_ (μM)	Tryptic peptide	Residue	Charge	MSc	XC	Δcn
ERK2	0.1	YTNLSYIGEGAYGMVC(O_3_)SAYDNLNK	Cys^38^	2	34	3.4	0.2
		YTNLSYIGEGAYGMVC(O_2_)SAYDNLNK	Cys^38^	3	NA	5.3	0.13
		SIDIWSVGC(O_2_)ILAEMLSNRPIFPGK	Cys^214^	2	NA	2.9	0.26
p38	20	DLKPSNLAVNEDC(O_3_)ELK	Cys^162^	3	63	3.2	0.27
JNK2	1	YQQLKPIGSGAQGIVC(O_3_)AAFDTVLGINVAVK	Cys^41^	2	98	5.1	0.67
		TLEEFQDVYLVMELMDANLC(O_3_)QVIHMELDHER	Cys^116^	3	54	NA	NA
		MSYLLYQMLC(O_3_)GIK	Cys^137^	2	31	1.4	0.2
		TAC(O_3_)TNFMMTPYVVTR	Cys^177^	2	35	2.8	0.41
		GC(O_3_)VIFQGTDHIDQWNK	Cys^222^	2	37	2.7	0.06

MAPK were oxidized with H_2_O_2._ Tryptic peptide fragment sequence, peptide charge, Mascot Ions Score (MSc), Sequest XC and Δcn value are shown. Δcn stands for the difference in the cross-correlation score between the top two candidate peptides or proteins for a given input data file. NA: not assigned. No oxidation was achieved with ERK2 at 10 μM H_2_O_2_, with JNK2 at 0.1 μM H_2_O_2_ or with p38 at <10 μM H_2_O_2_. (O_3_) sulfonic modification, (O_2_) sulfinic modification.

No JNK2 cysteines were oxidized with 0.1 µM H_2_O_2_, while 1 µM H_2_O_2_ oxidized the thiols of Cys^41^, Cys^137^, Cys^177^, and Cys^222^ to –SO_2_H, and Cys^116^ to –SO_3_H. It is remarkable that JNK2 Cys^41^, homologous to ERK2 Cys^38^, was sensitive to oxidation while JNK2 Cys^213^, homologous to ERK2 Cys^214^, was not oxidized at any H_2_O_2_ level. In contrast, p38 Cys^162^, homologous to an alternative docking domain of rat ERK1/2, was oxidized to –SO_3_H only after 20 µM H_2_O_2_ (Table 1). No methionine, histidine, or tryptophan oxidation, or tyrosine and tryptophan nitrosylation were detected.

### ERK-MEK interaction and ERK shuttle to nuclei depend on mitochondrial oxidation of the redox-sensitive cysteines

In order to assess the role of oxidizable cysteines on ERK activation and redistribution, ERK2 mutants C38A, C214A, and C214E were transfected onto LP07 cells to search for their interaction with MEK1/2. H_2_O_2_ oxidation enhanced wild type ERK2-MEK1/2 interaction (Fig. 8B), as was previously observed in Fig. 6B. However, H_2_O_2_ had no effect on the interaction of MEK1/2 with ERK2 when Cys^214^ was substituted by an Ala (C214A) (Fig. 8B). ERK2-MEK1/2 interaction was otherwise enhanced by the replacement of Cys^214^ with a Glu (C214E), even in the absence of H_2_O_2_ (Fig. 8B).

Wild type ERK2 translocated to mitochondria and was afterwards retained in nuclei after stimulation with 1 µM H_2_O_2_, as endogenous ERK (Fig. 8C and see Fig. 3). In contrast, ERK2 mutants C38A and C214A were retained in mitochondria in detriment of nuclear entrance (Fig. 8C). p-MEK1/2 was as well retained in mitochondria after transfection with ERK2 mutants C38A and C214A, which indicates that defective oxidation impedes ERK-MEK complex exit from mitochondria (Fig. 8D).

ERK redox-sensitive cysteine domains are well conserved in all MAPKs as well as in other kinases (Table 2). Noteworthy is the fact that both oxidable Cys^38^ in ERK2 and Cys^41^ in JNK2 are in the same domain and were oxidized to –SO_3_H, but at different H_2_O_2_ levels. JNK2 Cys^213^ and p38 Cys^48^ and Cys^231^ all present in these domains, were not oxidized.

**Table 2:** Redox sensitive cysteine domains.

JNK1	61	IGSGAQGIVCAAYD	JNK1	273	VDIWSVGCIMGEMV
JNK2	32	IGSGAQGIVCAA FD	JNK2	207	VDIWSVGCIMGLV
ERK2	31	EGAYGMVCAAYD	ERK2	207	IDIWSVGCIMGEMV
ERK1	51	EGAYGMVSAAYD	ERK1	227	VDIWSVGCILAEML
p38	40	GSGAYGSVCAAYD	p38	224	VDIWSVGCIMGEMV
1PME	91	GEGAYGIVCSAYD	1PME	229	IDIWSVGCIMGEMV
MMK1	51	IGHGAIGI VCSAHN	MMK1	185	IDIWSVGCIMGEMV
MPK1	20	GEGAYGIVCSAYD	MPK1	162	DVWSVGCIMGEMV
1TKIA	4	LGREFGIVCAAYD	1TKIA	193	TDIWSTGVIMGEMV
PHKA	3	LGRGVSVVCIH	PHKA	215	DVWSVGCIMGEMV

Alignment of homologue sequences corresponding to regions surrounding Cys^38^ and Cys^214^ of ERK2 in different kinases.

Aligment performed with Expasy Proteomics Server (www.ca.expasy.org). 1PME (penta mutant ERK2); MMK1 (mitogen-activated protein kinase homolog); MPK1 (mitogen-activated protein kinase); 1TKIA (human titin kinase); PHKA (human phosphorylase kinase subunit alpha).

## Discussion

In the present study we provide evidence that supports that H_2_O_2_ drives proliferation and cell cycle arrest by specific MAPKs activation in LP07 cells. Opposite effects elicited by low and high H_2_O_2_ concentrations have been previously observed in various tumor cell lines [10], as well as in normal tissues [6]; this supports the notion that increases in H_2_O_2_ concentrations change cell phenotype by eliciting sequential and opposite responses [3]. This process relies on the alternative activation of either ERK1/2 or JNK1/2 and p38 MAPKs by modulation of the interaction with their cognate MAPKKs. We also confirm the presence of MAPKs and MAPKKs in mitochondria and that these kinases redistribute to the cytosolic and nuclear compartments. The mechanism of MAPK import onto the outer membrane and intermembrane space still awaits elucidation [36]. MAPKs could enter and exit the organelle without previous mitochondrial damage, alike Bcl-2 or Bcl-x_L_, both mitochondrial proteins that translocate to nuclei [37], [38].

We show for the first time that modulation of MAPKs interaction with their upstream kinases relies on the oxidation of cysteines immersed in conserved domains of MAPKs (Table 2). A remarkable finding is that selective kinase activation is based on a differential sensitivity to oxidants, particularly H_2_O_2_. Furthermore, efficient ERK2 binding to MEK is achieved by oxidation of two of the five ERK2 cysteine thiols, Cys^38^ and Cys^214^, to cysteine sulfinic (-SOH_2_) and sulfonic (-SOH_3_) acid at very low H_2_O_2_. Interestingly, ERK2 Cys^38^ and Cys^214^ are not oxidized at high H_2_O_2_. A great variability in the oxidation of cysteine residues at high H_2_O_2_ concentration was previously found; this fact could be due to the molecular reorganization and competition with water, induced by the oxidant itself. Noteworthy is that JNK2 Cys^41^, homologous to ERK2 Cys^38^, was sensitive to oxidation while JNK2 Cys^213^, homologous to ERK2 Cys^214^, was not oxidized at any H_2_O_2_ level. On the other hand, p38 Cys^162^, homologous to an alternative docking domain of rat ERK1/2, was oxidized to –SO_3_H only at high H_2_O_2_ concentrations (20 µM).

ERK2 cysteine oxidation by H_2_O_2_ proceeds outside its catalytic site, increases binding to MEK1/2 by three to four-fold, modulates activity, and, more importantly, it does not occur at every H_2_O_2_ concentration, for it can only be achieved at low oxidant level. Cysteine post-translational modifications appear to be critical for the ERK activation pathway and kinase redistribution, and eventually in cell fate. Mutation of Cys^214^ to Ala rendered ERK2 and MEK1/2 accumulation in mitochondria, also observed under high H_2_O_2_ concentration.

Modulation of enzyme activity by cysteine oxidation to sulfenic acid has been reported: thiol oxidation has been shown to participate in the catalytic mechanism of peroxiredoxins (Prx) isoforms I-VI, which reduce H_2_O_2_ by forming disulphide [39], sulfenic acid (in Cys^51^ in mammals) [40], [41]. Prxs are further oxidized to sulfinic acid and inactivated [42], the same that MAPK phophatase-3 [43] and phosphatase-1B [44]. The cysteine sulfinic acid of Prxs can be reduced to cysteine by ATP-dependent sulfiredoxin [45] or sestrins-Hi95 or sestrin2 [46]. Together, these facts argue for the reversible oxidation of cysteines as a mechanism of protein activation.

**Figure 9:**
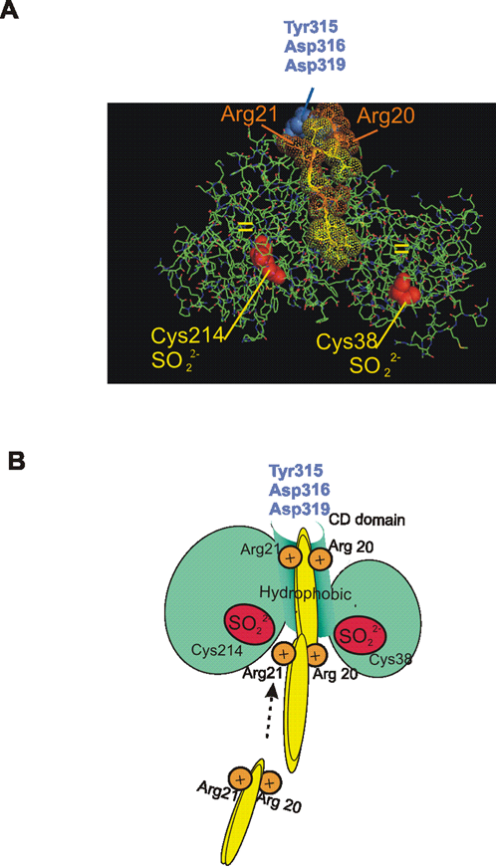
Scheme of electrostatic guide for ERK-MEK interaction based on cysteine charges. (A) MKP3-ERK2 interaction based on a crystallographic study (PDB 2GPH). MKP3 peptide is placed on ERK2 docking groove as a product of acidic and hydrophobic interactions with oxidizable cysteines (in red) (modelled with the PyMol program; DeLano Scientific, CA, USA). (B) Scheme based on (A) representing the ligand (yellow ovals) approaching ERK2 (green circles). The ligand “walks” through acidic charges, attracted by Cys^38^ and Cys^214^, to finally reach the common docking domain [54].

The domains surrounding the cysteines susceptible to oxidation in the former enzymes share an Arg residue (Cys+6 to Cys+11) [47] as well as those in MAPKs. Thiol oxidation requires a low p*K*a for the cysteine group [48]. When located in position Cys+10 or Cys+9, Arg helps decrease p*K*a below 5.8 (normal Cys p*K*a = 8.5), to dissociate Cys–SH to the thiolate (Cys–S^–^) at physiological pH, and to stabilize cysteine sulfinic oxidation. Because mutation of Cys^214^ to glutamic acid enhances ERK-MEK interaction, negative charges must be involved in the interaction and thus, we postulate that cysteine oxidation introduces negative charges with very low p*K*a (<2) that allow the interaction to occur [48]. An attractive idea is that charged cysteines lead MEK or other ligands as they “walk” through arginines to Asp^316^ and Asp^319^ (Fig. 9A and B), the two essential ERK acidic residues that integrate the D domain for binding to ligands [32]. p38 Cys^162^ negative charges might be attracted to the arginines of upstream ligands, like Arg^104^ of MKK3 [49]. With respect to JNK, the probable mechanism of redox binding is controversial, for oxidation proceeds in several Cys domains. This argues for the possibility that oxidation of multiple cysteines may stabilize the hydrophobic interactions involved in the binding capacity of proteins and also suggests that more than one model of regulation by oxidation could apply for the different biological systems and molecular pathways.

We conclude that proliferation of LP07 cells, and presumably in other cellular systems, depends on sustained oxidation of ERK2 thiols to sulfinic or sulfonic acid achieved at low oxidant yield. If dysfunctional mitochondria are incapable of increasing the oxidative level in cells [10], then they cannot contribute to cell cycle arrest either by oxidation of p38 Cys^162^ or by impeding Cys^38^ and Cys^214^ oxidation in ERK2 and, thus, drive to an uncontrolled cell division and ultimately to cancer.

## Materials and Methods

### Cell line, culture conditions and treatments

LP07 cell line was derived from P07 lung tumor spontaneously arisen in a BALB/c mouse, and extensively characterized [10], [50]. Cells were maintained in Dulbecco's modified Eagle's medium nutrient mixture F-12 HAM (D-MEM) with 10% fetal bovine serum (FBS) and 50 µg/ml gentamycin. For treatment, cells were 24 h serum starved and then stimulated with H_2_O_2_ and/or the MAPKs inhibitors (2 h prior to H_2_O_2_ treatment). When appropriate, cells were seeded onto a 22.1-mm diameter well and transiently transfected with wild-type 3XFlag-CMV7-ERK2 vector, the ERK2 mutants H230R and Y261N (kindly provided by Dr. M. Cobb) or ERK2 mutants C38A, C214A and C214E (Genscript, Piscataway, NJ, USA), with Lipofectamine 2000 (Invitrogen) according to manufacturer's instructions. After transfection, cells were stimulated and harvested, and subcellular fractions were recovered for western blot.

### Isolation of nuclear, mitochondrial and cytosolic fractions

Cells were lysed in MSHE buffer (0.22 M mannitol, 0.07 M sucrose, 0.5 mM EGTA, 2 mM HEPES/KOH, 1 mM phenylmethylsulfonylfluoride (PMSF), 5 µg/ml leupeptin, 5 µg/ml pepstatin, 5 µg/ml aprotinin, 25 mM NaF, and 1 mM sodium orthovanadate, pH 7.4). The homogenate was centrifuged 10 min at 1000× g (pellet = crude nuclear extract) and 20 min at 10,000× g (pellet = mitochondria; supernatant = cytosol). Mitochondria were resuspended in MSHE. The crude nuclear extract was washed with buffer A (10 mM Tris, 1.5 mM EDTA, 10% glycerol, 1mM PMSF, 5 µg/ml leupeptin, 5 µg/ml pepstatin, 5 µg/ml aprotinin, 5 mM NaF, and 1 mM sodium orthovanadate, pH 7.4) containing 0.01% NP-40, resuspended in buffer A plus 0.4 M KCl, and incubated 30 min at 4°C. The suspension was centrifuged 30 min at 105000× g and diluted with buffer A to reduce salt concentration. The purity of the fractions was assessed by western blot with antibodies against complex I or IV (mitochondria), by measuring lactate dehydrogenase activity (cytosol), and by flow cytometry with propidium iodide (nuclei) (data not shown). Protein content was determined by Lowry method.

### Western blot

Proteins separated by SDS-PAGE, were transferred onto PVDF membranes and immunoblotted with antibodies anti total and phosphorylated ERK1/2, p38, JNK1/2, MEK1/2, MKK3, MKK4 (Cell Signaling), cyclin D1 (Santa Cruz), complex I and IV (Molecular Probes) or Flag (Sigma), and then incubated with secondary antibodies conjugated with horseradish peroxidase (GE Health Care). Chemiluminiscence was detected with enhanced ECL reagent (GE Health Care).

### Proliferation assays

LP07 cells were seeded onto a 96-well plate (8.10^4^ cells/well) and treated as described above for 48 h in the presence of 0.8 µCi/well [^3^H] thymidine (specific activity, 70 to 90 Ci/mmol; NEN/Dupont, Boston, MA). Then cells were harvested and radioactivity measured in a liquid scintillation counter (Wallac 1414, Turku, Finland). Alternatively, LP07 cells seeded onto a 24-well plate (1.10^5^ cells/well) and treated as above, were harvested by trypsinization and counted in a Neubauer chamber in the consecutive days after stimulation.

### Cell growth assay

LP07 were seeded onto a 24-well plate, treated as above, and then harvested and counted in a Neubauer chamber in the consecutive days after stimulation.

### Cell cycle and apoptosis assays

Treated cells were harvested and incubated with (i) 100 µg/mL propidium iodide in 0.1% sodium citrate, 0.1% Triton X-100 at 4°C overnight in the darkness [51] or (ii) Annexin V-FITC (Immunotech) according to manufacturer's instructions. Cells were run on a FACScalibur flow cytometer (Becton-Dickinson, Mountain View, CA) and analyzed with WinMDI software for windows.

### Fluorescence labeling and confocal microscopy

Cells were grown on cover slides and stained with a specific mitochondrial marker, MitoTracker Deep Red 633 FM (Molecular probes) (100 nM, 45 min at 37°C), fixed in 4% paraformaldehyde, blocked in 1% BSA, 0.3% Triton X-100, PBS, pH 7.4, in a humidified chamber for 1 h, and incubated with primary (anti ERK1/2, JNK1/2 or p38) antibodies and secondary antibodies conjugated with Cy3 for 1h at RT in the same buffer. Cover slides were mounted in Fluorsave mounting media (Calbiochem). For *in vivo* imaging, cells were grown on Lab-Tek Chambered Borosilicate Coverglass System (Nunc), and transfected with GFP-hERK2 or JNK1-GFP, and stained with Mitotracker as described above. Confocal laser scanning microscopy was performed with an Olympus FV1000 using a 63×1.35 NA oil immersion objective. Excitation filters and emission detected with a PDA device were as follows: GFP, 488 nm excitation, 500–560 nm emission; Cy3, 532 nm excitation, 580±10 nm emission; MitoTracker Deep Red, 633 nm excitation, 650–750 nm emission. Images were acquired with Olympus Fluoview FV10-ASW software and analyzed with DIP image software for MATLAB (TNO, Delft). Images were analyzed by intensity correlation analysis (ICA) [51]; if two structures are part of the same complex or are present at the same place, then their staining intensities should vary in synchrony, whereas if they are on different complexes or structures they will exhibit asynchronous staining. ICA was done with MATLAB software as already developed, studied and reported [52]. Live images were analyzed as follows: a mask was done over the nucleus or over mitochondria when MitoTracker intensity was over 20, and GFP fluorescence was followed in these areas as well as in the whole cell. Nuclear and mitochondrial GFP fluorescence intensity was corrected for total photobleaching.

### Immune-electron microscopy

Cells were fixed in 4% paraformaldehyde and 0.5% glutaraldehyde in phosphate buffer 0.2 M pH 7.4 for 4 h. After washing in 0.1% glycine the samples were dehydrated in ethanol and embedded in LR White as described [6]. Samples were labeled overnight with anti p-ERK1/2 antibody diluted 1∶10, and then washed in 1% BSA and 0.05% Tween in PBS. Then they were incubated with a secondary antibody (1∶50) conjugated with 10 nm colloidal gold particles (Sigma anti-rabbit IgG) for 1 h at RT. Grids were washed, fixed in 2% glutaraldehyde for 10 minutes and counterstained in 2% uranyl acetate. Samples were analyzed on a Zeiss EM-C10 transmission electron microscope (50× and 100× objective) at 80k and photographed with 35 mm Kodak electron film.

### Co-immunoprecipitation assay

MAPKs were immunoprecipitated from cytosolic or mitochondrial fractions (500 and 300 µg of protein, respectively) in lysing buffer (50 mM Tris, 150 mM NaCl, 1 mM EDTA, 1 mM EGTA, 10% glycerol, 0.5% Nonidet P-40, 1 mM MgCl_2_, 1 mM PMSF, 5 µg/ml leupeptin, 5 µg/ml pepstatin, 5 µg/ml aprotinin, 25 mM NaF and 1 mM sodium orthovanadate, pH 7.4). Fractions were rocked for 2 h at 4°C and the immunocomplexes captured with protein A/G-agarose (Santa Cruz) or protein G-agarose (Sigma). Beads were washed in lysing buffer, boiled in loading buffer and run on SDS-PAGE.

### Kinase activity assay

*In vitro* phosphorylation assays were carried out without DTT [53]. GST-hERK2 was utilized and JNK1/2, p38 and the MAPKKs were immunoprecipitated in kinase buffer (20 mM HEPES, 2.5 mM MgCl_2_, 10 mM EDTA, 1% NP-40, 0.1% SDS, 40 mM β-glycerophosphate, 2 mM sodium ortovanadate, protease inhibitors, pH 7.5). Myelin basic protein (MBP) and ATF-2 were used as substrates for ERK2 or JNK1/2 and p38, respectively and phosphorylation was assessed by autoradiography. When appropriate, MAPKs were previously oxydized with 0.1–10 µM of H_2_O_2_ for 15 min. In mock experiments recombinant MAPKs kinases were absent.

### Pull down assay

Cytosolic or mitochondrial fractions were incubated with human recombinant ERK2-GST, p38-GST or JNK2-GST (Stressgen) oxidized with H_2_O_2_ and bound to agarose in lysing buffer for 2 h at 4°C. After incubation, agarose beads were washed in lysing buffer and cracked in loading buffer. Finally, samples were run on SDS-PAGE and detection of MAPKKs was assessed. To block the cysteines, proteins were incubated with 4-vinylpyridine (Sigma-Aldrich) for 1 h at RT, and then washed and oxidized. In mock experiments recombinant MAPKs kinases were absent.

### Mass spectrometry

Human ERK2, p38 or JNK2 proteins (Upstate) oxidized with 0.1–20 µM H_2_O_2_ were analyzed by mass spectrometry. We used the mutant non-phosphorylatable ERK K52R to avoid autophosphorylation and its putative effect on ERK oxidation. Tryptic digestion was performed with methylated trypsin to reduce autolysis (Promega, Madison, WI). Digestion products were dried in an APD SpeedVac (ThermoSavant), desalted by ZipTip (Millipore CB_18B_, Millipore, Billerica, MA) and resuspended in 60% acetic acid for injection by autosampler (Surveyor, ThermoFinnigan). Mass analysis was done in a ThermoFinnigan LCQ Deca XP Plus ion trap mass spectrometer equipped with a nanospray ion source (ThermoFinnigan). Proteins were identified with MS/MS Mascot (Matrix Science) search software using TurboSequest. Mascot and Sequest searches allowed for variable modifications as methionine, histidine or tryptophan oxidation (+16 Da) and cysteic acid reduction (+48 Da), and tyrosine and tryptophan nitrosylation (+45 Da). Sequest search also included variable mono and di-oxidation of cysteine (+16 and +32, respectively).

## Supporting Information

Figure S1 Redistribution of MAPKKs upon redox stimulation. Cellular distribution of (A) MEK1/2 (B) MKK4, and (C) MKK3 was followed as in Fig. 3. In (B) and (C), squares represent nuclei and circles represent mitochondria; red and black symbols indicate 1 and 50 µM H_2_O_2_, respectively. (D) On the bases of the experimental findings, a representative scheme proposes a cycle of MPKKs to transport cognate kinases from mitochondria to nucleus. Green circles indicate that MAPK are preferentially bound to upstream MAPKKs at the different redox states, respectively
(7.40 MB TIF)
